# Differential Expression of Neuroinflammatory mRNAs in the Rat Sciatic Nerve Following Chronic Constriction Injury and Pain-Relieving Nanoemulsion NSAID Delivery to Infiltrating Macrophages

**DOI:** 10.3390/ijms20215269

**Published:** 2019-10-24

**Authors:** Andrea M. Stevens, Lu Liu, Dylan Bertovich, Jelena M. Janjic, John A. Pollock

**Affiliations:** 1 Department of Biological Sciences, Bayer School of Natural and Environmental Sciences, Duquesne University, Pittsburgh, PA 15282, USA; steven10@duq.edu; 2 Chronic Pain Research Consortium, Duquesne University, Pittsburgh, PA 15282, USA; liul@duq.edu (L.L.); bertovichdt@jay.washjeff.edu (D.B.); Janjicj@duq.edu (J.M.J.); 3 Graduate School of Pharmaceutical Sciences, School of Pharmacy, Duquesne University, Pittsburgh, PA 15282, USA; 4 Washington and Jefferson College, Washington, PA 19610, USA

**Keywords:** chronic constriction injury, nanoemulsion, neuroinflammation, neuropathic pain, sciatic nerve, celecoxib, gene expression, neuroinflammatory mRNA

## Abstract

The neuroinflammatory response to peripheral nerve injury is associated with chronic pain and significant changes in the molecular expression profiles of mRNAs in neurons, glia and infiltrating immune cells. Chronic constriction injury (CCI) of the rat sciatic nerve provides an opportunity to mimic neuropathic injury and quantitatively assess behavior and differential gene expression in individual animals. Previously, we have shown that a single intravenous injection of nanoemulsion containing celecoxib (0.24 mg/kg) reduces inflammation of the sciatic nerve and relieves pain-like behavior for up to 6 days. Here, we use this targeted therapy to explore the impact on mRNA expression changes in both pain and pain-relieved states. Sciatic nerve tissue recovered from CCI animals is used to evaluate the mRNA expression profiles utilizing quantitative PCR. We observe mRNA changes consistent with the reduced recruitment of macrophages evident by a reduction in chemokine and cytokine expression. Furthermore, genes associated with adhesion of macrophages, as well as changes in the neuronal and glial mRNAs are observed. Moreover, genes associated with neuropathic pain including Maob, Grin2b/NMDAR2b, TrpV3, IL-6, Cacna1b/Ca_v_2.2, Itgam/Cd11b, Scn9a/Na_v_1.7, and Tac1 were all found to respond to the celecoxib loaded nanoemulsion during pain relief as compared to those animals that received drug-free vehicle. These results demonstrate that by targeting macrophage production of PGE_2_ at the site of injury, pain relief includes partial reversal of the gene expression profiles associated with chronic pain.

## 1. Introduction

Peripheral nerve injury underlying neuropathic pain affects over 100 million Americans and costs roughly $600 billion annually [1]. This type of injury can be modeled in small animals with chronic constriction injury (CCI) of peripheral nerves and is known to result in spontaneous pain-like behavior including amplified responses to painful stimuli (hyperalgesia), non-painful stimuli (allodynia) and thermal hypersensitivity [2,3,4,5]. Peripheral nerve injury is characterized by a complex molecular and cellular response, consisting of two main components. First, the injury is associated with a change in the immune cell microenvironment surrounding the damaged neurons leading to a local response known as neuroinflammation [6,7]. Second, healing and axon regeneration are engaged [8,9,10]. The initial immune response consists of infiltration of immune cells to the site of injury at the sciatic nerve, activation of immune and glial cells, and Wallerian degeneration of distal nerve fibers [11,12,13]. This process includes the recruitment of circulating monocytes to the site of injury, which release pro-inflammatory cytokines, chemokines, trophic and growth factors that cause neuronal hypersensitivity in the periphery [14]. The neurons themselves exhibit neuronal plasticity [15] leading to spontaneous firing, decrease in threshold, and an increase in responsiveness. Schwann cells along the injured axons dedifferentiate into an activated state where they switch from a myelinating phenotype to a phagocytic cell type, similar to macrophages. In addition, the injured neurons and Schwann cells produce and release inflammatory mediators [16] such as cytokines, chemokines, neurotrophic factors, Substance P, and prostanoids that all contribute to the neuroinflammatory milieu.

Monocyte-derived macrophages are known to play a role in peripheral nerve injury at the site of injury in the sciatic nerve and later at the level of the neuronal cell bodies in the dorsal root ganglia [5,17,18,19,20]. These hematogenous macrophages accumulate preferentially around injured axons [21] guided by molecular cues such as monocyte chemoattractant protein-1 (MCP-1) [22], the lesser studied monocyte chemoattractant protein-5 (MCP-5), and the leukocyte adhesion complex of MAC1, which is formed by the integrins, ITGAM/CD11b and ITGB2/CD18 [23]. Together with injured axons and activated Schwann cells, the infiltrating immune cells (macrophages, T cells, mast cells, and neutrophils) are crucial for normal function and recovery after nerve injury [17,24]. Each cell type appears to exhibit profiles of gene expression that contribute to the production of inflammatory mediators that further influence the neuropathic pain state [25,26,27,28]. Schwann cells at the site of injury through the distal nerve dedifferentiate by altering gene expression [25]. Within two days of chronic constriction injury, the Schwann cells stop producing myelinating proteins, and in turn, increase the expression of regeneration-associated genes such as GAP-43, neurotrophic factors and their receptors like GDNF, and cytokines such as IL-6 [13]. Hematogenous macrophages also produce the pro-inflammatory cytokine IL-6 and increase expression of cyclooxygenase 2 (COX-2), which produces prostaglandin E_2_ (PGE_2_), all of which contribute to the inflammatory response [18,29].

Janjic and co-workers demonstrated feasibility of targeting cyclooxygenase-2 (COX-2) enzyme in macrophages using cell specific delivery with near infrared labeled nanoemulsion (NE) loaded with non-steroidal anti-inflammatory (NSAID) drug, celecoxib (CXB) [30,31]. The celecoxib loaded nanoemulsion (CXB-NE), when administered to CCI rats, leads to significant reversal in pain-like behavior [5,30]. CXB-NE selectively inhibits COX-2 enzyme leading to dramatically reduced production of PGE_2_ in monocyte-derived macrophages at the site of injury [4,30,31,32]. Not only does the single dose of CXB-NE (0.24 mg/kg of celecoxib) provide pain relief for about 6 days, we have observed that at the site of injury there is a reduction in inflammation as visualized in live animals by near infrared fluorescence (NIRF), as well as histologically visualized reduction in the density of infiltrating macrophages at the site of injury [4,5]. Furthermore, for the macrophages that remain in the injured sciatic nerve even while treated with celecoxib-loaded nanoemulsion (CXB-NE), there is a reduction in PGE_2_ production [4,5] and a shift in M1 to M2 polarity [5]. We also see a reduction in mast cell numbers as well as a reduction in mast cell degranulation [5].

To further explore the molecular cell biology of the resultant chronic pain and the effects of the targeted celecoxib therapy, we assess mRNA expression in the injured sciatic nerve through quantitative PCR of a collection of 84 genes associated with neuroinflammation. In this study, we evaluate the molecular expression changes at the site of injury on day 12 post-CCI surgery, a time of significant pain-like hypersensitivity for untreated animals or maximum pain relief for treated animals. We compare the expression profile of mRNAs in the injured nerve to mRNA from comparable sciatic nerve acquired from sham surgical animals as well as CCI animals treated with nanoemulsion containing celecoxib (CXB-NE) or vehicle nanoemulsion that is drug free (DF-NE). We demonstrate significant changes in the neuroinflammatory mRNAs present within the injured sciatic nerve including genes associated with macrophages as well as neurons and Schwann cells among other cell types. Relative to vehicle (DF-NE), the mRNAs expressed in the drug treated (CXB-NE) exhibit decreased expression of seven known neuroinflammatory genes found in various cell types (Grin2b/NMDAR2b, IL-6, Itgam/Cd11b, Maob, Scn9a/Na_v_1.7, Tac1, TrpV3) and the increased expression of the neuronal Cacna1b/Ca_v_2.2. Many of the changes evident in the sciatic nerve that result from injury and the changes in response to pain reliving therapy may have their basis in regulated differential expression of key mRNAs. This research illustrates the need to better understand the molecular mechanisms involved in this neuroinflammatory-mediated multi-cellular response, and how by targeting only one inflammatory mediator, we are able to alter the expression of several genes while changing the pain-like behavior state.

## 2. Results

### 2.1. Relief of Pain Hypersensitivity in the CCI Model with a Drug-Loaded Nanoemulsion

**Figure 1:**
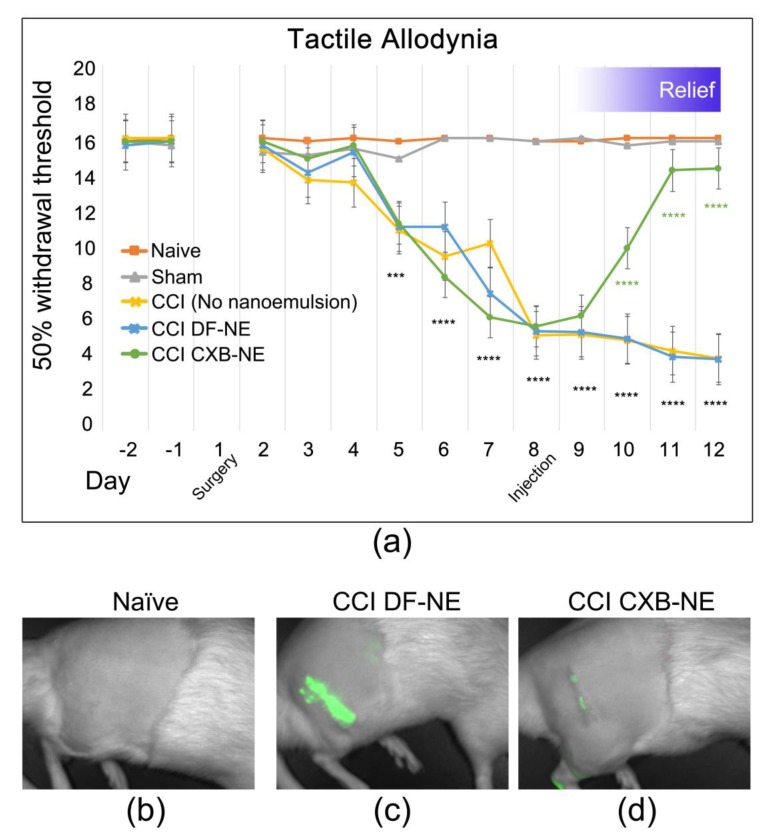
Celecoxib-loaded nanoemulsion (CXB-NE) ameliorates mechanical allodynia and inflammation in chronic constriction injury (CCI)-induced neuropathic male rats. (**a**) Mechanical stimulus evokes pain-like hypersensitivity in CCI animals. Treatment with CXB-NE provides a recovery from pain-like behavior comparable to sham and naïve control rats by day 12 (displayed as ****), but not in CCI rats given drug-free nanoemulsion (DF-NE). Data are expressed as a mean ± SD with a *n* = 5 per group. Significance was determined by two-way ANOVA followed by Tukey’s multiple comparison test (*p* < 0.0001). No statistical significance of difference of sham control compared to naïve, whereas the statistically significant difference is evident in the CCI animals compared to sham controls on days 5 through 8. (**b**–**d**) Live animal NIRF imaging on day 11 shows decreased fluorescence in CCI animals treated with CXB-NE (**b**), compared to those administered DF-NE (**c**). (**d**) Non-surgical naïve animals exhibit no fluorescence.

First demonstrated by Bennett and Xie [2] chronic constriction injury (CCI) of a peripheral nerve is known to induce hypersensitivity, which is interpreted as neuropathic pain caused by the neuroinflammation. Here we report that after baseline behavioral testing, CCI or sham surgery was performed on day 1, followed by behavioral testing daily on days 2 through 12 post-surgery. The effects of celecoxib-loaded nanoemulsion (CXB-NE) treatment on CCI rats was determined by using the paw withdrawal threshold when probed with von Frey filaments of increasing diameter [33,34]. All CCI animals experienced mechanical allodynia one-week post-injury, with a maximum in pain-like behavior plateauing on day 8 (Figure 1a), at which time celecoxib-loaded nanoemulsion (CXB-NE) or drug-free nanoemulsion (DF-NE) is administered by tail vein injection [35]. Data are expressed as the mean ± SD (*n* = 5 per group). Significance was determined by two-way ANOVA followed by Tukey’s post-hoc test (GraphPad Prism, version 7.3, San Diego, CA, USA). No statistically significant difference is evident in the comparison of sham control to the non-surgical naïve animal. Furthermore, no statistically significant difference was observed between CCI DF-NE animals compared to CCI animals receiving no nanoemulsion at all. A statistically significant difference in allodynia is evident between sham and naïve controls compared to CCI starting on day 5 post-surgery. One day after the nanoemulsion celecoxib drug therapy, treated animals exhibit a statistically significant reduction in hypersensitivity ultimately to return to a withdrawal latency equivalent to sham/naïve within three days of the tail vein injection. On day 12, the drug treated CCI animals exhibit a statistically significant relief in pain-like behavior as compared to both the CCI DF-NE (vehicle) and the CCI only (no nanoemulsion) animals (0.624, *p* value < 0.0001).

The nanoemulsion used in this study acts as both a therapeutic (delivering celecoxib) and diagnostic (loaded with a near infrared dye) [3,4,5,30,35,36,37]. Live animal near infrared fluorescent (NIRF) imaging on day 11 demonstrates increased inflammation at the site of injury (Figure 1c) as compared to naïve control animals (Figure 1b). CCI animals given the drug-loaded nanoemulsion (CXB-NE) exhibited a decreased NIRF signal as compared to DF-NE (Figure 1d), indicating a reduction in macrophage infiltration. Macrophages directly influence the milieu of inflammatory mediators such as cytokines and chemokines [38]. Although macrophages and other infiltrating immune cells accumulate at the site of injury post-CCI at day 12, neuronal-centric changes also contribute to the induction and maintenance of pain.

### 2.2. Overview of mRNA Expression Changes for Genes Associated with CCI Chronic Pain

**Figure 2:**
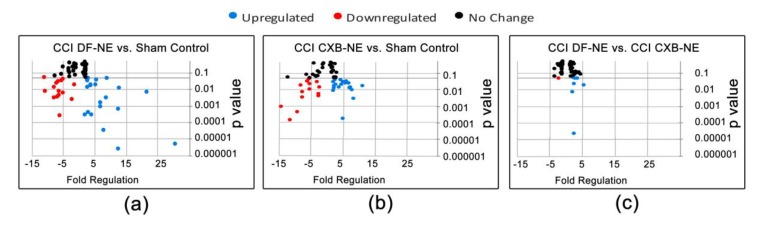
Neuroinflammatory gene expression changes in mRNA of sciatic nerve isolated on day 12 post-CCI injury, four days after nanoemulsion injection. In total, 84 genes associated with neuropathic and inflammatory pain were measured from Qiagen’s RT^2^ array (PARN-162Z). Qiagen’s RT^2^ profiler PCR Array Data Analysis software yielded differential expression of genes given as fold regulation in (**a**) CCI DF-NE compared to sham control, (**b**) CCI CXB-NE compared to sham, and (**c**) comparison of the two CCI conditions. Upregulated genes (blue), downregulated genes (red), and unchanged genes (black). *p* value < 0.05, 2-tailed Student’s *t*-test. The differential expression and *p* values for all genes are listed in Table 1.

While attenuating COX-2 activity at the site of injury through CXB-NE therapy, we examined the mRNA expression of 84 genes previously identified as associated with neuropathic and inflammatory pain in the rat (Qiagen, PARN162-Z, Germantown, MD, USA; Table S1). The dissected segment of the injured sciatic nerve includes axon fibers as well as myelinating Schwann cells and dedifferentiated Schwann cells, endothelial cells, and infiltrating macrophages among other activated immune cells. A quantitative analysis of the qPCR C_T_ values for the mRNAs identified by all 84 genes was carried out using Qiagen’s RT^2^ Profiler Array Software (version 3.5, Germantown, MD, USA) and displayed as a volcano plot between all data groups (Figure 2). This software creates a ‘volcano plot,’ which displays statistical significance versus fold change, by plotting *p*-value against the fold regulation change with the significance of the differential expression. Here, fold regulation values greater than 1 indicate increased gene expression, whereas fold regulation values less than zero indicate reduced expression.

In order to visualize gene expression changes of the entire 84 gene set, volcano plots were generated for each pairwise comparison (CCI DF-NE versus sham, CCI CXB-NE versus sham, and CCI DF-NE versus CCI CXB-NE). Illustrated in Figure 2, genes displaying statistically significant increased expression are shown in blue while reduced expression is indicated with red. Those that exhibit changes in expression but that are not statistically significant are shown in black. Because the printed graphic cannot support tags that name every data point, the differential expression of all the genes are listed in Table 1. Comparing CCI DF-NE animals (pain state) to the sham surgical control reveals that there were 24 of the 84 genes with increased expression and 16 genes with reduced expression (Figure 2a, Table 1a). In sham versus CCI CXB-NE animals (pain relieved), 19 genes showed increased expression and 14 exhibited decreased expression (Figure 2b, Table 1b). Comparing both CCI conditions (drug free to drug treated), there were 7 genes upregulated and 1 downregulated (Figure 2c, Table 1c).

Under the influence of immune cells, glia and the neuronal cell bodies in the corresponding dorsal root ganglia, the axons that make up the sciatic nerve exhibit exaggerated responses marked by hyperexcitability as well as changes in local expression of key mRNAs encoding products involved in hyperalgesia pain-like behaviors. For example, Calcitonin Gene-Related Peptide (CGRP) mRNA encodes a calcitonin peptide involved in the maintenance of neuropathic pain [39]. CGRP is significantly reduced in the CCI pain state, but not responsive to the CXB-NE drug therapy (CCI CXB-NE exhibited a fold change of 0.13; CCI DF-NE 0.12 when compared to sham). Similarly, the Neurotrophic receptor tyrosine kinase 1 (Ntrk1) mRNA is also reduced in the CCI pain state, and not responsive to the CXB-NE drug therapy as compared to sham (CCI CXB-NE fold change of 0.17 and CCI DF-NE fold change of 0.13). However, several mRNAs encoding ion channels exhibit significant differential expression in the sham surgical control versus the CCI celecoxib-loaded- (CXB-NE) and vehicle-loaded (DF-NE) nanoemulsions. These include Scn9a (Sodium voltage-gated channel, type IX or Na_v_1.7), TrpV3 (Transient receptor potential 3), and Cacna1b (Calcium voltage-gated channel alpha 1B or Ca_v_2.2).

Celecoxib selectively inhibits COX-2, blocking prostaglandin synthases [40]. Although induction of COX-2 occurs in multiple cells (Schwann cells, neurons, and macrophages), by directly targeting COX-2 and subsequent PGE_2_ production in only invading macrophages, we find that there is a reduction in PGE_2_ in the injured sciatic nerve [4,5]. In CCI CXB-NE, there is a marked increased expression in of Prostaglandin receptor 1 (Ptger1; fold change 2.29) and Prostaglandin E Synthase 2 (Ptges2; 2.21 fold change). However, there is no significant changes of expression for Prostaglandin receptors 3 and 4 (Ptger3 and Ptger4) when comparing the pain states with or without drug relative to sham control. Furthermore, no gene expression changes in any of the conditions were noted for other key mediators in the PGE_2_ pathway, including Prostaglandin E Synthase (Ptges), Prostaglandin E Synthase 3 (Ptges3), Prostaglandin-Endoperoxide Synthase 1/2 (Ptgs1 also known as COX-1 and Ptgs2 also known as COX-2).

When comparing CCI DF-NE (pain state) to sham, genes that exhibited increases in expression ranged from 1.68 fold for Gdnf to 30.5 fold for Mcp5, whereas downregulated expression ranged from −1.62 fold for Mapk3 to −11.03 Oprk1 (Figure 2a). In CCI CXB-NE (pain relief) animals versus sham, 19 genes showed increased expression and 14 exhibited decreased expression, ranging from a fold regulation of 11.02 in Mcp5 to −14.23 in Scn9a (Na_v_1.7) (Figure 2b). Comparing both CCI DF-NE to CCI CXB-NE conditions, there were seven genes with elevated expression in the pain state relative to the drug treated pain-relief state and one gene that exhibited reduced mRNA expression (Figure 2c).

### 2.3. Altered Expression of Key Mrnas Involved in Neuroinflammation

By day 12, four days after the nanoemulsion injection, the drug treated animals behave as the sham and naïve, exhibiting no hypersensitivity while the vehicle treated animals exhibit un-abated hypersensitive pain-like behavior (Figure 1a). Each of the neuroinflammatory genes differentially expressed in the array are listed in Table 2, Table 3, and Table 4 according to their cell-specific expression based on past pain-related studies in the literature. Each of the tables illustrates known roles for the described genes based on a literature review from PubMed. The differential RNA expression fold change described is from the data contained in this report for the analysis of the Neuropathic and Inflammatory pain RT^2^ array. Fold change of CCI CXB-NE rat sciatic nerve mRNAs relative to sham control and fold change of CCI DF-NE mRNAs relative to sham control were analyzed with Qiagen’s RT^2^ profiler PCR Array Data Analysis software, where statistically significant differentially expressed mRNAs were identified using a 2-tailed Student’s *t*-test, with a *p* value < 0.05, as calculated by the Qiagen software. Changes in gene expression between the pain group and the control group are illustrated as a fold increase/decrease and are considered to be upregulated/downregulated, respectively. Genes previously associated with expression in neurons, Schwann cells, circulating monocytes, macrophages, and T cells (citations noted in Table 2, Table 3 and Table 4) are found to be expressed at high levels in the pain state in the present study. Additionally, a superset of this data can be found in Table 1. Table 2 and Table 3 list differential expression revealing that eighteen of the 84 genes that were previously reported as exhibiting neuronal expression are affected by our treatments. Additionally, 6 genes are associate with immune cell, and another 15 of the differentially expressed mRNAs are typically found in multiple cell types that include Schwann cells, endothelial cells, and neuronal cells, all exhibiting elevated expression in the pain state.

**Figure 3:**
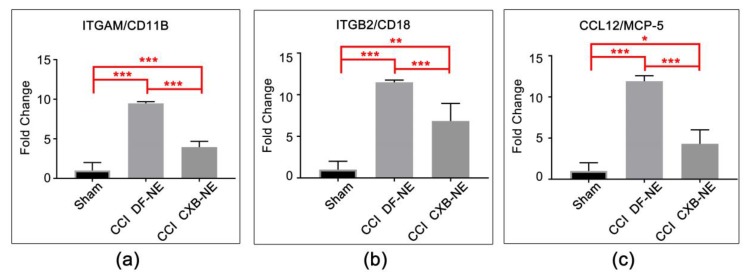
mRNA expression of macrophage-related genes at the site of injury. (**a**) Itgam/Cd11b mRNA is significantly elevated in the CCI pain-state (DF-NE) over sham, and is significantly reduced in the CXB-NE drug treated CCI condition. (**b**) Itgb2/Cd18 (integrin β-2) mRNA is significantly elevated in the CCI pain state (DF-NE) over sham, and is significantly reduced in the CXB-NE drug treated CCI condition. (**c**) Mcp-5 mRNA is significantly elevated in the CCI pain-state (DF-NE) over sham and is significantly reduced in the CXB-NE drug treated CCI condition. One-way ANOVA with Tukey post-hoc analysis is considered statistically significant if *p* value < 0.05. Sham = 6, CCI CXB-NE *n* = 8, CCI DF-NE *n* = 6. * *p* value ≤ 0.05; ** *p* value ≤ 0.001; *** *p* value ≤ 0.0001.

Ten of the differentially expressed genes that are revealed by the Qiagen RT^2^ Profiler Array software were re-analyzed with RNA from new animals utilizing individual qPCR assays with the same primers. In this way, the sample size was increased for each gene (*n* = 8–10 per condition). The genes chosen show statistically significant differential RNA expression in the Neuropathic and Inflammatory RT^2^ array analysis and are known to be involved in the neuroinflammatory response. Macrophage-associated genes analyzed included Itgam/Cd11b (Figure 3a), Itgb2/Cd18 (Figure 3b), and Mcp5/Ccl12 (Figure 3c). ITGAM/CD11b and ITGB2/CD18 form the integrin MAC-1 complex, which is responsible for the recruitment and adhesion of leukocytes to the site of injury [23,59]. The role of chemokine MCP-5 (monocyte chemotactic protein 5) in neuropathic pain has not been studied in the CCI model but is thought to behave like MCP-1, which is involved in the recruitment of monocytes/macrophages to injured tissue [60].

**Figure 4:**
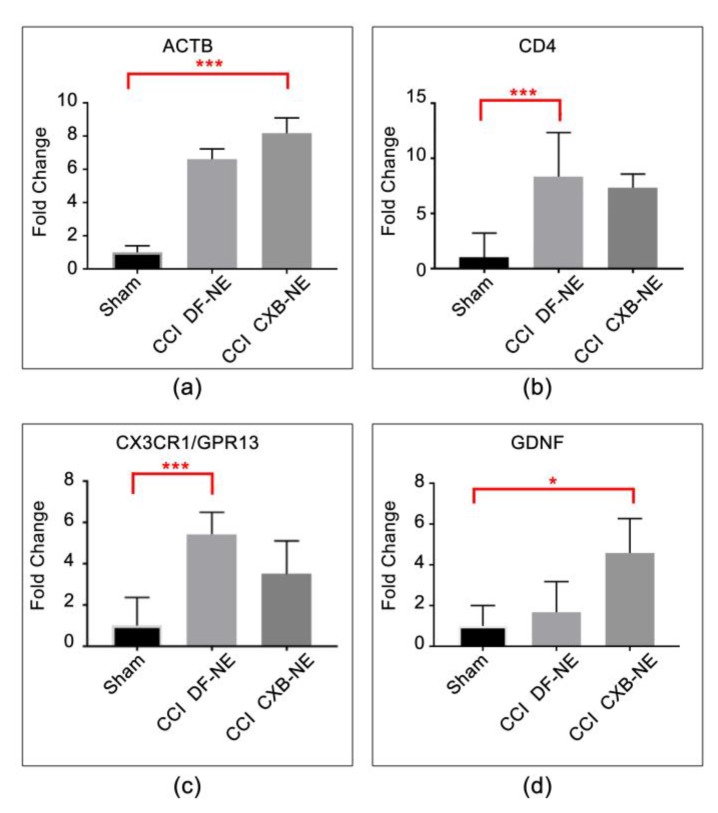
mRNAs responsive to CCI. (**a**) Actin beta (Actb) mRNA expression is elevated in response to CCI treatment and is not responsive to CXB-NE drug therapy. (**b**) Cd4 is typically associated with T cells and is similarly responsive to CCI treatment but is not responsive to CXB-NE therapy. (**c**) Cx3cr1 (Chemokine CX-3-C motif, receptor 1) is expressed widely on the cell surface of neurons, macrophages, circulating monocytes, T cells, and Schwann cells. The mRNA expression is elevated in response to CCI treatment. (**d**) Gdnf (glial-derived neurotrophic factor) is produced by neurons and glial cells and appears to be responsive in the CCI condition to the CXB-NE drug therapy. One-way ANOVA with Tukey post-hoc analysis is considered statistically significant if *p* value < 0.05. Sham = 6, CCI CXB-NE *n* = 8, CCI DF-NE *n* = 6. * *p* value ≤ 0.05; *** *p* value ≤ 0.0001.

**Figure 5:**
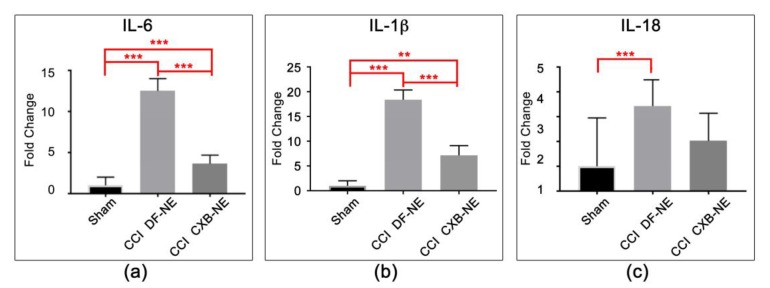
Cytokine mRNA expression at the site of injury. (**a**) cytokine IL-6 (interleukin-6) mRNA is significantly increased in response to CCI and is significantly attenuated with CXB-NE drug therapy. (**b**) IL-1β (interleukin-1 beta) mRNA is significantly increased in response to CCI, and is significantly attenuated with CXB-NE drug therapy. (**c**) IL-18 (interleukin-18) are produced and released by multiple cell types at the site of CCI injury is significantly increased in response to CCI, and is attenuated with CXB-NE drug therapy. One-way ANOVA with Tukey post-hoc analysis is considered statistically significant if *p* value < 0.05. sham = 6, CCI CXB-NE *n* = 8, CCI DF-NE *n* = 6. ** *p* value ≤ 0.001; *** *p* value ≤ 0.0001.

Other differentially expressed genes include Actb, Gdnf, Cd4, Cx3cr1, IL-1β, IL-6 and IL-18. These expression profiles are associated with multiple cell types including neurons, Schwann cells, monocytes/macrophages, and T cells (Table 2, Table 3 and Table 4). β-actin and Cd4 (Figure 4a,b) are significantly increased in expression in the CCI condition relative to sham, but their expression level is not sensitive to the drug therapy CXB-NE treatment. Similarly, Cx3cr1/Gpr13 is increased in its mRNA expression in the CCI conditions relative to sham. While the Cx3cr1/Gpr13 in the drug treated CXB-NE condition is reduced relative to the DF-NE, the reduction is not statistically significant in this experiment (Figure 4c). Interestingly, Gdnf is significantly increased in its mRNA expression in the drug treated CXB-NE CCI condition (Figure 4d). IL-6, IL-1β and IL-18 (Figure 5) all exhibit significantly increased mRNA expression in the CCI (DF-NE, pain-state) as compared to sham and that they are also each responsive to the drug therapy. No expression was seen in sham animals.

### 2.4. Differential Expression of mRNAs in the CCI (Pain State) Compared to Celecoxib-Treated CCI (Pain Relieved)

**Figure 6:**
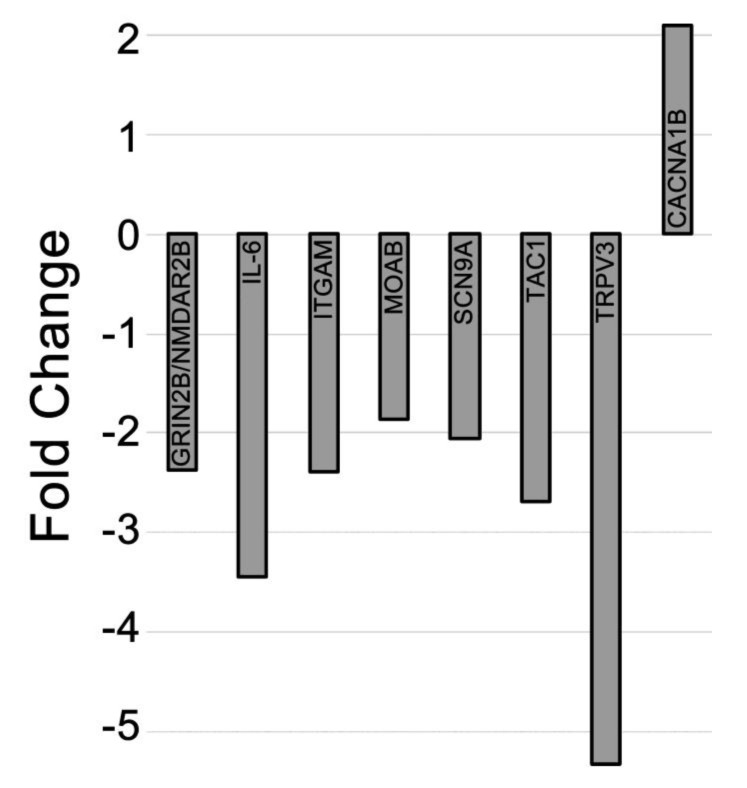
mRNA differential expression comparing CCI CXB-NE relative to CCI DF-NE animals. CCI animals given CXB-NE exhibit decreased expression of 7 known neuroinflammatory genes found in various cell types in the pain state and increased expression of the axonal Cacna1b gene when compared to CCI DF-NE animals.

Genes that exhibited differential expression of mRNAs in the CCI conditions were compared to the mRNA expression levels in the drug treated condition (Table 1c). This helps to reveal genes where their expression is directly affected by CXB-NE COX-2 inhibition in monocyte-derived macrophages in the sciatic nerve. Differential expression of the mRNAs between the CCI pain state and drug-treated pain state shows that seven genes are down-regulated and one gene is upregulated in the treated animals (Figure 6). Maob expression is elevated in the CCI pain-state (DF-NE) and exhibits a downregulation in CCI CXB-NE. The pro-inflammatory mediator interleukin-6 (IL-6) mRNA expression is primarily produced by Schwann cells, neurons, circulating monocytes and macrophages [18] and exhibits an elevated pattern of expression that is decreased when CXB-NE is present. Itgam/Cd11b is expressed by circulating monocytes and macrophages [23] and shows a 2-fold reduction in expression when circulating monocytes are targeted with celecoxib treatment in the CCI state. Grin2b/NMDAR2b, Scn9a/Na_v_1.7, Maob, Tac1 and TrpV3 are all exhibiting increased expression in the pain state, while reduced when CXB-NE provides relief and are each previously associated with neuronal expression [55,57,58,61,76]. In contrast, the voltage-dependent calcium channel, Cacna1b, is significantly increased in CCI CXB-NE animals compared to CCI DF-NE.

### 2.5. Identification of CD68 and CD11b Macrophages within the Site of Injury

**Figure 7:**
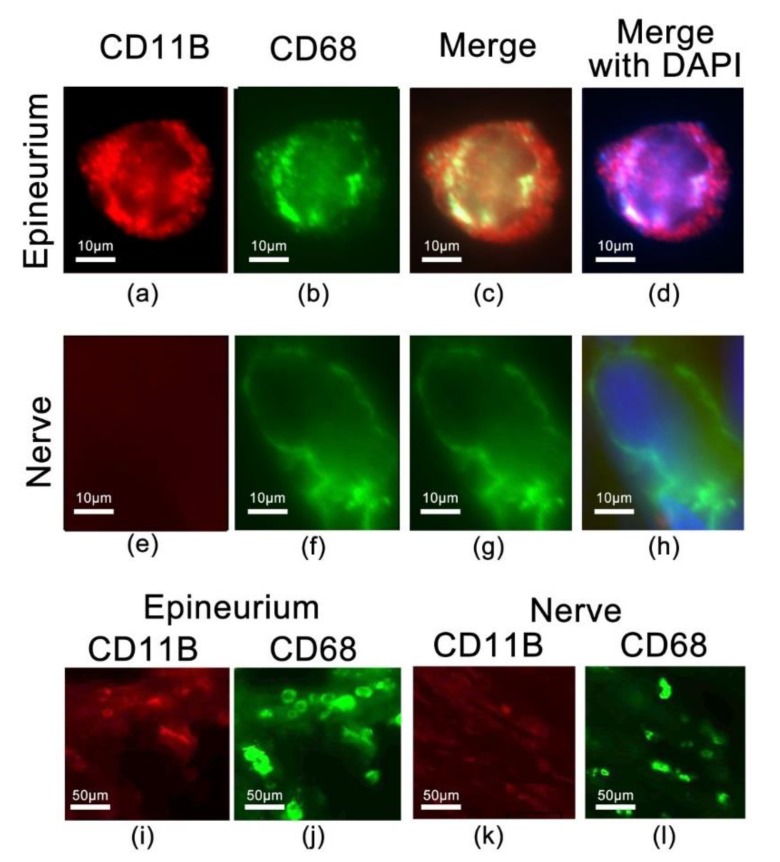
A subset of CD68 positive macrophages co-express the integrin ITGAM/CD11b, detected with anti-CD11b antibody. Co-expressing cells are predominantly found in the epineurium (**a**–**d**), whereas the CD68 positive macrophages in the nerve track do not co-express CD11b (**e**–**h**). (**i**,**j**) reveals the co-expressing CD11b and CD68 cells predominate in the epineurium, but only CD68 positive cells are prominent in the nerve (**k**–**l**).

Our previous studies show a decrease in the number of CD68 macrophages that infiltrate the injured nerve under the same conditions using CXB-NE [4,5]. Anti-CD68 is a pan macrophage marker that has the ability to identify macrophages that are both phagocytic as well as cells that are actively producing and releasing cytokines and chemokines, whereas anti-CD11b is associated with more specific macrophages [78]. We find that the CD11b-positive cells also co-express CD68 in the pain state, but that not all CD68 positive cells are CD11b positive (Figure 7). CD11b-positive macrophages tend to be small and round in nature (Figure 7a–d), where CD68-positive macrophages can also be large, foamy, and irregularly shaped (Figure 7e–h). CD68 positive macrophages are found predominantly within the injured sciatic nerve, with low expression of CD11b positive macrophages (Figure 7k,l), whereas CD11b/CD68 positive macrophages are found primarily in the epineurium surrounding the nerve fibers (Figure 7i,j). These findings are consistent with previous literature of neuropathic pain models showing protein expression of both CD11b positive and CD68 positive macrophages with immunohistochemistry [19,78,79]. Parallel downregulation of CD11b macrophages is consistent with the reduction in expression of inflammatory mediators such as IL-6, IL-18, and IL-1β as noted by their mRNA expression.

## 3. Discussion

### 3.1. Peripheral Nerve Injury is Associated with Differential Expression of Neuroinflammatory mRNAs

This study reveals that mRNA expression changes are seen directly at the site of injury where neuroinflammation is rampant. Furthermore, that within 4 days of celecoxib-loaded nanoemulsion administration, aside from a reduction in pain-like behavior and reduction in PGE_2_ production, shift in macrophage polarity and reduction in Mast Cell degranulation [4,5], we now find that the regulated expression of several mRNAs are responsive to the drug-therapy. This drug-induced differential expression of mRNAs for key neuroinflammatory genes is a result of the nanoemulsion drug being delivered to macrophages in the pain state.

Overall, our data reveals changes in the mRNA expression of genes associated with neuroinflammation, which is consistent with phenotypic behavioral changes as demonstrated by the assessment of tactile allodynia of the affected hind leg. CCI animals demonstrate no significant allodynia during the initial Wallerian degeneration phase occurring between days 1 to 3 [27] and show an increased pain threshold during days 4 to 5 and beyond [4,5]. By day 8 post-surgery, we observe a plateau in hypersensitivity that persists in un-treated animals [4,5]. However, following intravenous injection of celecoxib-loaded nanoemulsion on the eighth day post-surgery, we show that beginning as early as a day later, there is a significant reduction in mechanical allodynia, consistent with prior observations [4,5]. We have previously demonstrated that this single dose (0.24 mg/kg) of celecoxib provides relief from hypersensitivity that lasts about 6 days [4,5]. This represents greater than a 2000-fold reduction in dosage that would be needed were traditional daily oral dosing utilized [80]. This change in behavioral phenotype overlaps with the prolonged neuroimmune response occurring in and around the site of injury. This is characterized by the changes in behavioral activity and activation of resident immune and immune-like glial cells [26,81,82]. In vivo imaging of the ipsilateral hindleg revealed an increased near infrared signal consistent with an accumulation of nanoemulsion-labeled macrophages at the site of injury, indicative of neuroinflammation associated with infiltrating macrophages [3,4,5]. Immunohistochemical investigation of dissected sciatic nerves reveals that CCI CXB-NE animals on day 12 exhibit a reduced infiltration of macrophages as compared to CCI DF-NE animals [4,5]. COX-2 as well as with PGE_2_ are present in the injured rat sciatic nerves following CCI injury [4,5] suggesting injured nerve-derived invading macrophages may influence the neuroimmune cellular environment within the sciatic nerve through possible autocrine and paracrine pathways [19]. Here we show that the heightened and persistent neuroimmune response in the peripheral nerve injury leads to changes in mRNA expression at the site of injury, and that it involves multiple cell types, including neurons, macrophages, Schwann cells, degranulating Mast cells and T cells; all of which contribute to the production of inflammatory mediators at the site of injury.

### 3.2. Targeting COX-2 in Macrophages Contributes to a Decrease in Macrophage-Related mRNAs at the Site of Injury

By directly targeting COX-2 enzyme and PGE_2_ production in invading macrophages at the site of injury, there is a subsequent decreased gene expression evident in Cd11b/Itgam and Cd18/Itgb2 beta-2 integrins when compared to sham control. Cd11b/Itgam and Cd18/Itgb2 are integrins that heterodimerize to form a complex known as MAC-1 or CR3. It is normally present in an inactive confirmation in circulating monocytes, but is quickly activated to mediate leukocyte adhesion, migration and accumulation of cells at the site of inflammation [23]. Previous studies have shown that by blocking MAC-1 and its’ ligands or the ablation of its genes encoding Cd11b or Cd18, leads to a decrease in severity of inflammation in animal models [23]. Given the attenuation in COX-2 activity and the reduction in PGE_2_ production in CXB-NE targeted macrophages [4], the differential expression in Cd11b between CCI animals given drug versus drug-free CCI animals supports the observation that fewer macrophages are attracted to the site of injury. Here, we hypothesize that by inhibiting COX-2 and the production of PGE_2_ in circulating monocytes and tissue macrophages, there is a subsequent change in macrophage signaling, including integrins, cytokines, and the involvement of other cell types that participate in releasing inflammatory mediators at the site of injury that leads to decreased pain behaviors when administered at a chronic pain state in rats. Additionally, macrophages functions are dynamic with a shift in polarity when given celecoxib-loaded nanoemulsion [5]. Our lab has previously shown there is a nearly 30% reduction of M1 macrophages in the ipsilateral sciatic nerve of pain treated (CXB-NE) animals on day 12 [5]. Temporally, there is multitude of surface markers used to identify macrophages after nerve injury [82]. A study by Lee and Zhang [78] showed that in injured nerves, macrophages exhibit heterogeneity, where a subpopulation identified by the MAC1 (CD11b/CD18) marker is cytokine/chemokine expressing and another subpopulation labeled by CD68 antibody is predominantly phagocytic (which is key for the regeneration process). Previously, our studies have shown that CD-68 positive macrophages are present in high number within the damaged nerve (CCI DF-NE) compared to CCI animals receiving CXB-NE [4,5]. We also demonstrated that the CD68 positive macrophages in the injured sciatic nerve can be distinguished as M1 (pro-inflammatory) and M2 (anti-inflammatory) by their expression of the costimulatory protein, Cluster of Differentiation 40 (CD40), or transferrin receptor (TFRC) respectively [5]. Utilizing the cytokine/chemokine secreting-specific antibody to CD11b, we are now able to further identify additional phenotypically different macrophages in the CCI nerve. In a review by Ristoiu et al. (2013), they found that in the sciatic nerve there is activation of CD11b-positive and CD68-positive macrophages after several neuropathy-induced animal models [19]. Our observation here is that at this moment in time (day 12 post CCI surgery), CD68-positive macrophages are found predominantly within the injured sciatic nerve, whereas CD11b/CD68-positive macrophages are found primarily in the epineurium surrounding the nerve fibers.

### 3.3. COX-2 Inhibition in Macrophages Leads to a Reduction in Pro-Inflammatory Cytokine mRNA

Previous studies suggest that macrophage-derived PGE_2_ alters neural activity, influences the milieu of cytokines, and changes gene expression at the site of injury, all of which contribute to the chronic pain state [3,4,5,18,67,83,84,85,86]. Nerve injury results in a significant increased expression of IL-6 in injured nerves [16,18,87]. IL-6 is also elevated in invading macrophages in vivo [18], ensheathing Schwann cells and increased in T cells in response to possible increased PGE_2_ expression [75]. IL-6 is a pleiotropic cytokine where an increased production can be neurotrophic for neurons and can provide further signals that influence changes in neuronal and glial cell gene expression [75]. Consistent with previous studies [72], we found that IL-6 mRNA expression is elevated post-CCI in the ipsilateral sciatic nerve. Expression of IL-6 also plays a role in axonal regeneration when overexpression of the protein and its receptor results in improved nerve regeneration [88,89].

The mRNA expression for cytokines IL-18 and IL-1β is increased in CCI DF-NE animal’s sciatic nerve, with IL-1β responding to CXB-NE treatment, showing decreased expression when nanoemulsion drug therapy is used. This is consistent with the observation that both IL-18 and IL-1β exhibit elevated expression at the protein level in the sciatic nerve of CCI rats, and that the activation of the inflammasome combined with the dominant role exhibited by IL-18 during chronic inflammation contributes to chronic pain [38].

Tachykinins are a family of conserved peptides encoded by the Tac1 gene, which generates substance P, neurokinin A, neuropeptide δ, and neuropeptide γ [90,91]. These neuropeptides are mainly expressed in neuronal tissues in the central nervous system and in primary afferent neurons in the peripheral nervous system [91]. It has also been shown that substance P and neurokinin A are expressed in non-neuronal cells including macrophages and circulating monocytes [91]. Substance P is a neurotransmitter that has been implicated in chronic pain [92]. NMDA receptors like GRIN2b/NMDAR2 are present in primary afferents and evoke the release of substance P from their central terminals [92]. Interestingly, mRNAs encoding both substance P and GRIN2b/NMDAR2 are differentially expressed in the injured sciatic nerve and are responsive to CXB-NE. This suggests that the transcriptional regulation of GRIN2b/NMDAR2 as well as Tac1 mRNAs play a role in regulating pain transmission and that when COX-2 is attenuated in macrophages their co-regulation is similarly reduced.

### 3.4. mRNAs are Differentially Expressed in Pain Relief versus the Untreated Pain State

When comparing differential expression between the hypersensitive pain state (DF-NE) and the pain-relieved state (CXB-NE), there are 8 of the 84 genes that exhibit differential expression; five of which are known to be expressed in neurons [56,93,94]. Of these five, Grin2b/NMDAR2, Scn9a/Na_v_1.7, and Maob are expressed in neurons [55,76,93] and there is an increase in the mRNA expression for these five genes in the injured sciatic nerve during hypersensitivity. Furthermore, when COX-2 activity is attenuated by the targeted delivery of nanoemulsion loaded with celecoxib, there is a reduction in this over-expression. Changes in Na_v_1.7/Scn9a, Maob, and Cacna1b mRNA expression in chronic pain is thought to be involved in the maintenance of chronic pain in the peripheral nervous system [95,96,97]. The role of Trpv3 is not well understood in peripheral neuropathy though its expression has been reported in mouse peripheral nervous systems [56] and the mRNA exhibited high expression levels in rat DRG in spared nerve injury models of neuropathic pain [98].

### 3.5. mRNAs at the Site of Injury Contributing to Anti-Inflammation and Regeneration

The neurotrophic factor GDNF is known to have neuroprotective function in L4 and L5 DRG neurons after axotomy [99]. Here we see increased expression of GDNF between sham surgical sciatic nerve and CCI nerve treated with CXB-NE. GDNF is a survival promoting factor for nociceptive neurons and has been shown to have analgesic properties when spinal nerve ligation injured mice are injected with GDNF-expressing lentivirus [100], suggesting GDNF mRNA have an anti-inflammatory role under neuropathic conditions. We also observe that β-actin (Act-β), which is involved in growth cone formation [41,101], exhibits differential mRNA expression. Interestingly, β-actin is often and mistakenly used as a “housekeeping gene” with the thought that its expression would remain unchanged after most nerve injuries. Our study revealed that, in fact, β-actin mRNA expression is altered upon CCI injury, with increased expression evident in CCI DF-NE animals. Drug-treated animals (celecoxib) exhibit a significantly higher level of expression over what is evident in the DF-NE condition. This increase in actin expression may be in response to the activation of peripheral nerve regenerative processes [102].

Here we report that the chemokine MCP-5 appears to play a role in neuropathic pain. MCP-1 is known as one of the main proteins that activates and attracts circulating monocytes to the injured nerve [22]. MCP-5 is similar to MCP-1 and is known to attract circulating monocytes and T cells [60]. Expression of MCP-5 can be induced by macrophages and possibly Schwann cells, but its overall role in neuropathic remains unclear. Our study shows a dramatic increase in MCP-5 mRNA in the pain state in the damaged sciatic nerve. When animals are given intravenous injection of CXB-NE, there is a significant reduction in MCP-5 expression, indicating a role for this gene at the site of injury in the CCI peripheral nerve.

### 3.6. Neuroinflammatory Genes Previously Associated with Cell Bodies Are Differentially Expressed at the Site of Injury

Exploration of differential expression of neuroinflammatory genes in previous studies has focused on changes in the dorsal root ganglia (DRG) and the dorsal horn. The chemokine, fractalkine, is thought to be present only in DRG neurons during the neuropathic pain response [103] with its receptor being expressed on a wide variety of cell types, including neutrophils, monocytes, CD8 T cells, Mast Cells, Natural Killer cells, fibroblasts, neurons, and endothelial cells [14,68]. Interactions within the neuronal–glial–immune-cell triad results in amplification of sensory signals so that responsivity to noxious as well as innocuous stimuli is intensified in ways that contribute to the pain-like behavior [68]. Verge and colleagues [68] studied the fractalkine mRNA, showing that it is upregulated in DRG neurons as a result of CCI with a marked increase in expression of its G-Protein Coupled receptor, CX3CR1, in microglia as well as in neurons in the spinal cord. Here, we find that the mRNA expression of CX3CR1 is significantly increased in the periphery in the injured sciatic nerve in the drug-free nanoemulsion compared to sham. When treated with nanoemulsion loaded with celecoxib, the elevated expression of CX3CR1/GPR13 is reduced. Others have shown that when CX3CR1 is depleted by Diphtheria toxin treatment in CD11b-positive macrophages, 3 days following spinal nerve transection, no hypersensitivity was noted. By one week post-nerve injury, macrophages repopulated with no pain behavior evident [14]. When these same mice undergo the same nerve injury, neuropathic pain developed, suggesting that repopulated macrophages are able to engage in the development of inflammation leading to pain-like behavior [14].

In conclusion, the peripheral nerve response to injury involves complex responses from multiple cell types and it is for this reason we sought to profile mRNA expression for a specific set of genes involved in neuroinflammation by using mRNA derived from the site of injury. Our examination focused on the expression of genes involved in pain response modulation including those associated with inflammation, neurotransmitters signaling, neurotrophins, purinergic receptors, cytokines, and chemokines as well as products associated with the conduction of pain including ion channels, sodium channels, potassium channels, signal transduction/transcription, and synaptic transmission. We find differential expression of mRNAs in several of these categories in the pain-state as well as genes that are subsequently responsive to CXB-NE nanoemulsion drug therapy. Furthermore, analyzing gene expression in the sciatic nerve offers insight into how mRNA expression distal to the neuronal cell bodies may be of significant importance in establishing and maintaining chronic pain.

## 4. Materials and Methods

### 4.1. Animals

Adult male Sprague–Dawley rats (Hilltop Animals, Springdale, PA, USA) weighing 225–250 g at the time of surgery were used in all protocols that were performed in accordance with the guidelines outlined in the Guide for the Care and Use of Laboratory Animals of the National Institutes of Health and the regulations of the Institutional Animal Care and Use Committee (IACUC) at Duquesne University and the approved protocol #1501-01 (31 January 2015). Animals were acclimated to standard living conditions and kept on a 12-hour light, 12-hour dark cycle and given food and water ad libitum. Animals were socially housed, kept on paper bedding, and given special diet (Research Diets, Inc, New Brunswick, NJ, USA; catalog #AIN-93G) to avoid autofluorescence during imaging. Efforts were made to minimize the number of animals used and the time experiencing pain. The sample size was calculated based on power analysis of our previous work.

### 4.2. Peripheral Nerve Injury

Chronic constriction injury (CCI) was performed [2,3,5] as it emulates peripheral neuropathic pain by causing intraneural edema at and around the common sciatic nerve. Briefly, animals were anesthetized using respiratory isoflurane. All surgical procedures were performed under aseptic conditions. The biceps femoris and gluteus superficialis muscles were exposed and separated on the hindleg, followed by exposure and isolation of the sciatic nerve. Four chromic gut sutures were loosely ligated around the common sciatic nerve, separated by 1 mm gaps. The muscle and skin layers were then closed. In this procedure, chromic gut sutures contribute to the neuroinflammatory response. In sham control animals, the common nerve is exposed and isolated, but no sutures were used. Naïve controls received no surgical intervention.

### 4.3. Behavioral Testing

Mechanical allodynia testing was conducted one day prior to surgery and the following days post-operatively, days 3, 4, 5, 8, 11, and 12. Testing was performed in naïve, sham, CCI CXB-NE, and CCI DF-NE rats using the up-down method as described previously [5,33,34]. Calibrated Semmes-Weinstein von Frey monofilaments were applied to the plantar surface of the right and left hind paws in the region innervated by the common sciatic nerve. The monofilaments were applied in ascending order of gram force ranging from 0.41 to 15.13 gram force. The 50% paw withdrawal threshold was calculated using the median lethal dose (LD50), and treatment groups were analyzed by two-way ANOVA and Tukey’s post-hoc test [5].

### 4.4. Nanoemulsion Preparation and Delivery

The celecoxib loaded nanoemulsion (CXB-NE) and the drug-free nanoemulsion (DF-NE) [36,37] were injected intravenously through the right lateral tail vein on day 8 post-surgery [35]. Rats were lightly anesthetized with isoflurane and given 0.24 mg/kg of celecoxib nanoemulsion (or drug-free vehicle) using a 27-gauge needle. By near infrared imaging of the tail vein before and after injection, we can establish whether the injection was optimal (injected directly into the tail vein and little residual fluorescence is shown) or suboptimal (injected subcutaneously with a high degree of fluorescence in the tail) [35]. When injected into the bloodstream, the nanoparticle is phagocytosed by circulating monocytes and naturally delivered to the site of injury, which has been confirmed in previous studies, where those cells differentiate into tissue macrophages [3,4,5].

### 4.5. NIRF Imaging

On day 11 post-surgery, live NIRF imaging was conducted on lightly anaesthetized (1.5% Isoflurane) rats. The left and right hindlegs were imaged on the LiCOR Pearl Impulse Small Animal Imager (Lincoln, NE, USA). Near infrared dye accumulates at the site of injury superficial to the site of incision on the hindleg as it picked up by monocyte-derived macrophages [3]. Images are acquired in the 700 nm channel and a white light channel and analyzed with LiCOR Image Studio Lite (version 5.0, LiCOR Biosciences, Lincoln, NE, USA) as based on previous studies [3]. A hand-drawn region of interest (ROI) is drawn over the sciatic nerve region on the hindleg and relative fluorescence is calculated (total fluorescence divided by area within the ROI).

### 4.6. Tissue Dissection

Rats were euthanized by CO_2_ asphyxiation in a chamber on day 12. Right and left sciatic nerves were dissected immediately upon euthanasia. Sciatic nerve tissues for immunohistochemistry were post-fixed in 4% paraformaldehyde (PFA), moved to 0.4% PFA after 24 hours, and within 1 week, placed in a 30% sucrose 1× PBS solution before being mounted in OCT medium and cut longitudinally in 20 µm sections. Sciatic nerve tissue for RNA studies were immediately transferred to RNAlater (Life Technologies, New York, USA) and kept at −20 °C until RNA extraction was performed.

### 4.7. RNA Extraction and cDNA Conversion

Total RNA was extracted from intact ipsilateral and contralateral nerves using the RNAeasy Plus Mini Kit (Qiagen, Germantown, MD, USA) according to the kit’s instructions. Tissue disruption and homogenization was done with a mortar and pestle and then with QiaShredder columns (Qiagen, Germantown, MD, USA). RNA quality and concentration was assessed on the NanoDrop 1000 Spectrophotometer (Thermo Fisher Scientific, Delaware, USA) and was determined by running a sample with RNA loading dye (Ambion, Austin, TX, USA) on a 1% agarose gel and inspecting for distinct 18S and 28S bands, indicating lack of degradation. Quantity was determined by A260/A280 measurement. All samples had A260/A280 ratios of 1.9−2.3. Total RNA was used for complimentary DNA conversion by the RT^2^ First Strand Kit (Qiagen, Germantown, MD, USA). Three to six animals per condition were used.

### 4.8. Quantitative PCR and Analysis

RT^2^ RNA Quality Control PCR Array (Qiagen, PARN-999Z) was used to assess each sample prior to gene expression profiling. Gene expression profiling was analyzed by RT^2^ Rat Neuropathic and Inflammatory Pain Arrays (Qiagen, PARN-162Z) on the ABI StepOnePlus cycler (Applied Biosystems, Foster City, CA, USA) with RT^2^ SYBR green with ROX (Qiagen, Germantown, MD, USA) according to manufacturer’s instructions. Each array contains 84 genes related to neuropathic pain and inflammation (Table S1), 5 housekeeping genes, and 7 controls. Each array was performed in triplicate and normalized to Rplp1 (Figure S1). Data was initially analyzed with Qiagen’s RT^2^ profiler PCR Array Data Analysis software. A list of differentially expressed genes was identified using a 2-tailed Student’s *t*-test. Changes in gene expression between the pain group and the control group were illustrated as a fold increase/decrease (shown as fold regulation) and were considered to be upregulated/downregulated, respectively. The criteria were a *p* value *<* 0.05 and a mean difference equal to or greater than 2-fold.

Selected genes were further analyzed by individual RT^2^ primer assays (Qiagen) following the Livak method [104]. Fold change values were calculated compared to the sham control group. A 2-tailed Student’s *t*-test, standard deviation and error were calculated. The results were expressed as means with standard deviations. For the significance test, a one-way analysis of variance followed by post hoc testing and Tukey’s multiple comparison test with GraphPad Prism (version 7.3, San Diego, CA, USA). A *p* value ≤ 0.05 was considered statistically significant in all analyses.

### 4.9. Immunohistochemistry and Microscopy

Sciatic nerves from naïve, CCI CXB-NE, and CCI DF-NE rats (3 per condition) were used for immunohistochemistry to explore the CD68 macrophage (Abcam, Cambridge, UK; ab125212, 1:100), and CD11b (Life Technologies, Carlsbad, CA, USA; MA181606, 1:100) macrophage infiltration. Tissue sections were post-fixed in 4% PFA solution in 1× PBS, permeabilized with 0.3% Triton X-100 solution, blocked with 1× PBS normal donkey serum, and stained with primary and secondary antibodies. The sections were washed and incubated overnight with secondary 1:200 Alexa Fluor antibodies (Invitrogen, Carlsbad, CA, USA) and then washed and mounted with Prolong Gold DAPI nuclear stain (Invitrogen). All stained sections were viewed on the Nikon Eclipse Ni-U microscope (Melville, NY, USA) and images were acquired with the Nikon NIS-Elements software.

## 5. Conclusions

By attenuating COX2 activity specifically in infiltrating macrophages, there is a subsequent change in expression of mRNAs associated with neuroinflammation in the injured sciatic nerve. Based on previous gene–cell localization studies, this change in expression is not limited to macrophages, but also genes associated with other cell types at the site of injury (neurons, Schwann cells, T cells, and endothelial cells). The products of some of these genes are known to contribute to increased infiltration of immune cells, activation of immune-like glial cells at the site of injury, and the production of inflammatory mediators as well as influence axonal regeneration. Furthermore, mRNAs generally associated with the cell bodies in the DRGs such as the calcium channel genes Cacna1b and TrpV3 are found to be differentially expressed in the injured sciatic nerve. Overall, with the utilization of celecoxib-loaded nanoemulsion, this targeted inhibition of COX2 and reduction PGE_2_ in macrophages in turn influences gene regulation and expression throughout the sciatic nerve after peripheral nerve injury in male rats.

## Figures and Tables

**Table 1:** Gene list representing volcano plots in Figure 2. Expression changes are represented as fold regulation. Positive values indicate an increased expression, whereas negative values indicate decreased expression. (**a**) Genes differentially expressed in CCI DF-NE versus sham, (**b**) genes differentially expressed in CCI CXB-NE versus sham, (**c**) genes differentially expressed in CCI DF-NE versus CCI CXB-NE.

(a). CCI DF-NE vs. Sham.					
Upregulated. CCI DF-NE			Downregulated CCI DF-NE		
Gene	Fold Regulation	*P* Value	Gene	Fold Regulation	*P* Value
Actb	6.61	0.0008	Cacna1b	−6.35	0.006
B2m	1.75	0.0002	Calca	−7.91	0.002
Adrb2	4.97	0.017	Cckbr	−6.67	0.02
Ccl12/Mcp5	30.5	>0.00001	Grm1	−6.67	0.02
Ccr2	2.41	0.03	Grm5	−6.42	0.004
Cd4	8.34	0.003	Kcnj6	−7.16	0.02
Cnr2	2.68	0.045	Kcnq3	−5.76	0.03
Comt	2.73	0.0004	Maob	−6.18	0.0002
Cx3cr1	12.47	0.0006	Mapk3	−1.62	0.016
Ednra1	3.59	0.018	Ntrk1	−7.88	0.012
Gch1	3.76	0.0002	Oprd1	−5.03	0.005
Gdnf	1.68	0.059	Oprk1	−11.03	0.048
Il−18	6.57	0.001	Opr1m	−10.85	0.007
Il−1α	8.81	0.04	Penk	−5.21	0.03
Il−1β	21.52	0.006	Ptger3	−2.19	0.002
Il−6	12.5	0.01	Scn9a	−6.89	0.003
Itgam/Cd11b	12.12	>0.00001			
Itgb2/Cd18	11.49	>0.00001			
P2rx4	2.93	>0.00001			
P2rx7	3.5	0.016			
Prok2	5.42	0.03			
Ptger4	2.39	0.013			
Tlr2	12.22	>0.00001			
Tnf	7.76	>0.00001			
Actb	6.61	0.0008			
B2m	1.75	0.0002			
**(b). CCI CXB-NE vs. Sham.**					
**Upregulated** **CCI CXB-NE**			**Downregulated** **CCI CXB-NE**		
**Gene**	**Fold Regulation**	***P*** **Value**	**Gene**	**Fold Regulation**	***P*** **Value**
Actb	8.18	0.002	Cacna1b	−3.03	0.02
B2m	1.84	0.009	Calca	−7.5	0.003 0.013
Adrb2	4.9	0.02	Edn1	−2.25	0.007
Alox5	2.38	0.03	Grm5	−7.64	0.02
Ccl12/Mcp5	11.02	0.017	Il−2	−5.22	0.04
Ccr2	2.58	0.02	Kcnq3	−5.7	0.00013
Cd4	7.35	0.01	Maob	−11.54	0.003
Comt	1.89	0.03	Mapk3	−2.47	0.003
Cx3cr1	7.78	0.009	Ntrk1	−5.98	0.03
Gdnf	4.51	0.02	Oprd1	−5.31	0.01
Il-18	4.27	0.04	Oprm1	−8.02	0.01
Il-1β	6.06	0.03	Ptger3	−2.46	0.005
Il-6	3.63	0.013	Scn9a	−14.23	0.0009
Itgam/Cd11b	5.06	0.0001	Trpv3	−9.12	0.0004
Itgb2/Cd18	6.84	0.02			
P2rx4	2.28	0.01			
Ptger1	2.29	0.01			
Tlr2	7.12	0.01			
Tnf	5.64	0.02			
**(c). CCI DF-NE vs. CCI CXB-NE.**					
**Upregulated** **CCI DF-NE**			**Downregulated** **CCI DF-NE**		
**Gene**	**Fold Regulation**	***P*** **Value**	**Gene**	**Fold Regulation**	***P*** **Value**
Grin2b	2.37	0.021	Cacna1b	−2.1	0.04
Il-6	3.44	0.04			
Itgam/Cd11b	2.39	>0.00001			
Maob	1.87	0.007			
Scn9a	2.06	0.05			
Tac1	2.69	0.04			
Trpv3	5.33	0.01			
Grin2b	2.37	0.021			

**Table 2:** Differential expression of mRNAs typically associated with neurons.

Gene Symbol	Gene Description	Fold Change Relative to Sham for CCI DF-NE	Fold Change Relative to Sham for CCI CXB-NE	GenBank	Citation
Actb ^1^	Beta-actin	6.61	8.18	NM_031144	[41]
Cacna1b/Ca_v_2.2	Calcium channel, voltage-dependent, N type, alpha 1B subunit	0.16	0.33	NM_147141	[42]
Calca/CGRP	Calcitonin-related polypetide alpha	0.12	0.13	NM_017338	[43,44]
Comt	Catechol-O-methyltransferase	2.73	1.89	NM_012531	[45,46]
Ednra	Endothelin receptor type A	3.59	0.44	NM_012550	[47]
Grm1	Glutamate receptor, metabotropic 1	0.15	Not detected	NM_017011	[48]
Grm5	Glutamate receptor, metabotropic 5	0.16	0.13	NM_017012	[48]
Kcnj6	Potassium inwardly-rectifying channel, subfamily J, member 6	0.14	Not detected	NM_013192	[49,50]
Kcnq3	Potassium voltage-gated channel, KQT-like subfamily, member 3	0.17	0.18	NM_133322	[49,50]
Ntrk1/TrkA	Neurotrophic tyrosine kinase receptor, type 1	0.13	0.17	NM_021589	[51,52]
Oprd1	Opioid receptor, delta 1	0.2	0.19	NM_012617	[53]
Orpk1	Opioid receptor, kappa 1	0.09	Not detected	NM_017167	[53]
Oprm1	Opioid receptor, mu 1	0.09	0.12	NM_013071	[53]
P2rx4	Purinergic receptor P2X, ligand-gated ion channel 4	2.96	2.28	NM_031594	[54]
Scn9a/Na_v_1.7	Sodium channel, voltage-gated, type IX, alpha	0.15	0.07	NM_133289	[55]
TrpV3	Transient receptor potential cation channel, subfamily V, member 3	Not detected	0.11	NM_001025757	[56,57,58]

^1^ Denotes genes individually analyzed by qPCR.

**Table 3:** Differential expression of mRNAs typically associated with immune cells.

Gene Symbol	Gene Description	Fold Change Relative to Sham for CCI DF-NE	Fold Change Relative to Sham for CCI CXB-NE	Cell Expression	GenBank	Citation
Adrb2	Adrenergic beta-2 receptor	4.97	4.9	Macrophages, Circulating monocytes	NM_012492	[61]
Ccr2	Chemokine (C-C) receptor 2	2.41	2.58	Macrophages	NM_021866	[62,63]
Cd4 ^1^	CD4 cell	8.34	7.35	T Cells	NM_012705	[64]
Cnr2	Cannabinoid receptor 2	2.68	Not detected	Macrophages	NM_012784	[65,66]
Itgam/Cd11b ^1^	Integrin, alpha M	12.12	5.06	Macrophages, Circulating monocytes	NM_012711	[59]
Itgb2/Cd18 ^1^	Integrin, beta 2	11.49	6.84	Macrophages, Circulating monocytes	NM_001037780	[59]
Mcp5/Ccl12 ^1^	Chemokine (C-C motif) ligand 12	30.5	11.02	Macrophages	NM_001105822	[60]
Ptgs2	Prostaglandin E synthase 2	Not detected	2.21	Macrophages, Circulating monocytes	NM_001107832	[67]

^1^ Denotes genes individually analyzed by qPCR.

**Table 4:** Differential expression of mRNAs typically associated with multiple cells in the periphery.

Gene Symbol	Gene Description	Fold Change Relative to Sham for CCI DF-NE	Fold Change Relative to Sham for CCI CXB-NE	Cell Expression	GenBank	Citation
Cx3cr1 ^1^	Chemokine (C-X3-C motif) receptor 1	12.47	7.78	Macrophages, circulating monocytes, neuronal, T cells	NM_133534	[16,68]
Gch1	GTP cyclohydrolase 1	3.76	Not detected	Macrophages, neuronal, T cells, mast cells	NM_024356	[69]
Gdnf ^1^	Glial cell derived neurotrophic factor	1.68	4.51	Neuronal, Schwann cells	NM_019139	[70]
IL-18 ^1^	Interleukin 18	6.57	4.27	Macrophages, circulating monocytes, neuronal, Schwann cells	NM_019165	[38,71]
IL-1β ^1^	Interleukin 1 beta	21.52	6.06	Macrophages, circulating monocytes, neuronal, endothelial cells	NM_031512	[72,73,74]
IL-6 ^1^	Interleukin 6	12.5	3.63	Macrophages, circulating monocytes, Schwann cells	NM_012589	[72,75]
Maob	Monoamine oxidase B	0.16	0.09	Neuronal, Schwann cells, endothelial cells	NM_013198	[76]
Mapk3	Mitogen-activated protein kinase 3	0.62	0.40	Neuronal, Schwann cells	NM_017347	[39]
Ptger1	Prostaglandin E receptor 1	Not detected	2.29	Macrophages, neuronal	NM_013100	[77]
Ptger3	Prostaglandin E receptor 3	0.46	0.41	Macrophages, neuronal	NM_012704	[77]
Ptger4	Prostaglandin E receptor 4	2.39	2.14	Macrophages, neuronal	NM_032076	[77]
Tlr2	Toll-like receptor 2	12.22	7.12	Macrophages, circulating monocytes, Schwann cells, T cells	NM__198769	[13]
Tnf	Tumor necrosis factor	7.76	5.64	Macrophages, circulating monocytes, Schwann cells, T cells	NM_012675	[72]

^1^ Denotes genes individually analyzed by qPCR.

## Supplementary Materials

Supplementary materials can be found at https://www.mdpi.com/1422-0067/20/21/5269/s1.

## Abbreviations

CCI	Chronic constriction injury
NIRF	Near infrared fluorescence
CXB-NE	Celecoxib-loaded nanoemulsion
DF-NE	Drug-free nanoemulsion (vehicle)
