# Supplementary material for: Differential Expression of Neuroinflammatory mRNAs in the Rat Sciatic Nerve Following Chronic Constriction Injury and Pain-Relieving Nanoemulsion NSAID Delivery to Infiltrating Macrophages
Source: Int J Mol Sci. 2019 Oct 24;20(21):5269. doi: 10.3390/ijms20215269 (PMC6862677; doi:10.3390/ijms20215269)
Supplement: Supplementary file 1 [file ijms-20-05269-s001.pdf]

Differential Expression of Neuroinflammatory Mrnas in the Rat Sciatic Nerve Following Chronic Constriction Injury and Pain-Relieving Nanoemulsion Nsaid Delivery to Infiltrating Macrophages

Andrea M. Stevens, Lu Liu, Dylan Bertovich, Jelena Janjic and John A. Pollock

Table S1. Gene list of RT[2] Profiler Array: Neuropathic and Inflammatory Rat Pain (Qiagen). These genes have been implicated in the transduction, maintenance, and modulation of pain responses after tissue damage.

Ace	Cd200	Gch1	Il2	Mapk8	Pla2g1b	Scn11a
Adora1	Cd4	Gdnf	Il6	Ngf	Pnoc	Scn3a
Adrb2	Chrna4	Grin1	Itgam	Ntrk1	Prok2	Scn9a
Alox5	Cnr1	Grin2b	Itgb2	Oprd1	Ptger1	Slc6a2
Bdkrb1	Cnr2	Grm1	Kcnip3	Oprk1	Ptger3	Tac1
Bdnf	Comt	Grm5	Kcnj6	Oprm1	Ptger4	Tacr2
Cacna1b	Csf1	Htr1a	Kcnq2	P2rx3	Ptges	Tlr2
Calca	Cx3cr1	Htr2a	Kcnq3	P2rx4	Ptges2	Tlr4
Cck	Dbh	Il10	Maob	P2rx7	Ptges3	Tnf
Cckbr	Edn1	Il18	Mapk1	P2ry1	Ptgs1	Trpa1
Ccl12	Ednra	Il1a	Mapk14	Pdyn	Ptgs2	Trpv1
Ccr2	Faah	Il1b	Mapk3	Penk	Scn10a	Trpv3

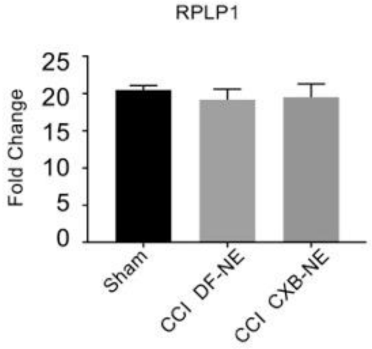

Figure S1. Normalization of genes to the housekeeping gene, Rplp1. Average CT value ranged from 18.5 to 20.1 in all samples. One-way ANOVA was not statistically significant amongst sham control, CCI drug-free (vehicle)-loaded nanoemulsion (DF-NE), or CCI celecoxib-loaded nanoemulsion (CXB-NE)
